# Periprocedural complications after laser balloon ablation procedures for atrial fibrillation: An analysis using a nationwide claims database

**DOI:** 10.1016/j.hroo.2025.07.016

**Published:** 2025-07-29

**Authors:** Ken Kawase, Reina Tonegawa-Kuji, Koshiro Kanaoka, Yoko Sumita, Koji Miyamoto, Kengo Kusano, Yoshihiro Miyamoto

**Affiliations:** 1 Department of Cardiovascular Medicine, National Cerebral and Cardiovascular Center, Suita, Osaka, Japan; 2 Department of Advanced Cardiovascular Medicine, Graduate School of Medical Sciences, Kumamoto University, Kumamoto, Kumamoto, Japan; 3 Department of Medical and Health Information Management, National Cerebral and Cardiovascular Center, Suita, Osaka, Japan; 4 Open Innovation Center, National Cerebral and Cardiovascular Center, Suita, Osaka, Japan

**Keywords:** Laser balloon ablation, Atrial fibrillation, Cryoballoon ablation, Radiofrequency ablation, Periprocedural safety

## Abstract

**Background:**

Evidence regarding the periprocedural safety of laser balloon (LB) catheter ablation for atrial fibrillation (AF) is scarce.

**Objective:**

We aimed to investigate the periprocedural safety of LB ablation for AF compared with that of either radiofrequency or cryoballoon ablation using a nationwide claims database.

**Methods:**

Patients who underwent catheter ablation for AF between 2018 and 2022 were identified using the Japanese Registry of All Cardiac and Vascular Diseases - Diagnosis Procedure Combination. The outcomes included in-hospital mortality, emergency 30-day readmission, common periprocedural complications, and a composite of complications. Outcomes were compared between the treatment groups in 2 separate propensity score–matched cohort: LB vs radiofrequency groups and LB vs cryoballoon groups. Mixed-effects logistic regression analysis was performed to calculate the odds ratios for the outcomes.

**Results:**

Overall, 179,207 patients who underwent AF ablation were included. The incidence rate of periprocedural complications after LB ablation was 3.2%, including a 1.6% incidence of stroke. In propensity score–matched cohorts, LB ablation procedures were associated with a higher incidence of periprocedural complications compared with radiofrequency ablation procedures. Furthermore, LB ablation was associated with a higher incidence of stroke or transient ischemic attack than those after cryoballoon or radiofrequency ablation. No significant differences were observed in in-hospital deaths, other complications, or emergency readmissions between LB ablation and either radiofrequency or cryoballoon ablation.

**Conclusion:**

LB ablation was associated with a higher incidence of stroke compared with either cryoballoon or radiofrequency ablation. Most patients experiencing stroke after LB ablation were discharged home.

**Key Findings**

▪ This study includes the largest cohort to date of patients undergoing atrial fibrillation ablation with the laser balloon (LB).
▪ The overall complication rate for LB ablation was 3.2%, and there were no significant differences in the incidence of cardiac tamponade, emergency readmission, or in-hospital mortality compared with other ablation modalities.
▪ LB ablation was associated with a significantly higher incidence of perioperative stroke (1.6%) compared with radiofrequency or cryoballoon ablation.
▪ Stroke was the most common complication after LB ablation; however, 86% of the affected patients were discharged home, suggesting that the events were generally mild.

## Introduction

Catheter ablation (CA) is recommended for symptomatic patients with recurrent paroxysmal atrial fibrillation (AF) or those with persistent AF resistant to previous treatment with antiarrhythmic drugs.^1^ The laser balloon (LB; HeartLight, CardioFocus Inc.) is a deflectable, balloon-shaped device designed to enable pulmonary vein isolation (PVI) with direct visualization at the left atrial–pulmonary vein junction.^2^^,^^3^ Once the target location and direct contact with the tissue are confirmed using an endoscope and a system called the “arc generator,” 980-nm laser energy is applied to the designated site. In a randomized controlled trial comparing LB and radiofrequency ablation for paroxysmal AF, the safety and efficacy of LB ablation were noninferior to radiofrequency ablation.^4^ Additionally, LB ablation demonstrated similar efficacy and safety in wide-area circumferential PVI to that using radiofrequency ablation.^5^ Compared with cryoballoon ablation, some studies have reported similar periprocedural safety of LB ablation, although these findings are based on small patient populations.^6^, ^7^, ^8^, ^9^, ^10^, ^11^, ^12^ However, the periprocedural safety of LB ablation has not been reported in large populations within a real-world setting.

The Japanese Registry of All Cardiac and Vascular Diseases - Diagnosis Procedure Combination (JROAD-DPC) is a nationwide claims database that uses data from the Japanese DPC/Per Diem Payment System.^13^, ^14^, ^15^, ^16^ In this study, using this nationwide database, we aimed to clarify the periprocedural safety of LB ablation for AF compared with cryoballoon or radiofrequency ablation.

## Methods

### Data source

This retrospective cross-sectional study used the JROAD-DPC database, which has been previously described in detail.^13^, ^14^, ^15^, ^16^ The JROAD-DPC database is an administrative database that covers >800 hospitals in Japan, most of which are Japanese Circulation Society–certified training hospitals. It includes the following information for each patient: age, sex, body mass index (BMI), main diagnoses and comorbidities, drugs, diagnostic and therapeutic procedures, length of stay, unique hospital identifiers, and discharge status.^17^ The DPC database contains 6 categories of diagnoses based on the *International Classification of Diseases, Tenth Revision* codes, each with a limited number of recordable diseases. One diagnosis each is coded for “main diagnosis,” “admission-precipitating diagnosis,” “most resource-consuming diagnosis,” and “second most resource-consuming diagnosis.” A maximum of 4–10 diagnoses each can be coded for “comorbidities present at time of admission” and “conditions arising after admission.” The CHA_2_DS_2_-VASc score was calculated for every patient using a point system in which 2 points are assigned for a history of stroke or transient ischemic attack (TIA) or age ≥ 75 years. One point was assigned for age between 65 and 74 years; a history of hypertension, diabetes mellitus, heart failure, or vascular disease (prior myocardial infarction or peripheral artery disease); and female sex.

This study was approved by the Institutional Review Board of the National Cerebral and Cardiovascular Center (R23004; April 9, 2023). The requirement for informed consent was waived because information specific to individuals was not included in the database. The study was conducted in accordance with the Declaration of Helsinki.

### Study population

**Figure 1:**
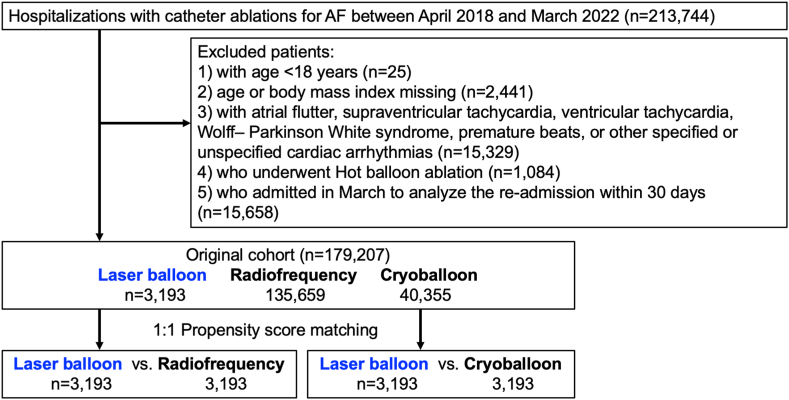
Flowchart of the study. AF = atrial fibrillation.

A flowchart of the study is shown in Figure 1. We identified 213,744 hospitalizations with CA for AF between April 2018 and March 2022. The diagnostic and procedural codes used are summarized in Supplemental Table 1. To facilitate the comparison between LB ablation and cryoballoon or radiofrequency ablation, the following patients were excluded: (1) aged < 18 years (n = 25); (2) age or BMI missing (n = 2441); (3) with other arrhythmias, to avoid inclusion of patients who had ablation procedures for other arrhythmias, as performed in previous analyses of CA for AF (n = 15,329)^18^, ^19^, ^20^, ^21^; (4) who underwent hot balloon ablation (n = 1084); or (5) who were admitted in March to analyze readmission within 30 days (n = 15,658). Consequently, 179,207 patients (LB: n = 3193; radiofrequency: n = 135,659; cryoballoon: n = 40,355) were included in this study.

### Outcomes

We identified the common in-hospital complications attributable to CA for AF using *International Classification of Diseases, Tenth Revision* diagnosis and DPC procedure codes. In-hospital complications were extracted from diagnoses coded for “conditions arising after admission” or procedure/device codes for those used after CA. The combined complications were a composite of all complications (stroke or TIA, spasm of the coronary artery, sick sinus syndrome, complete atrioventricular block, myocardial infarction, phrenic nerve palsy, pneumothorax, hemothorax, pneumonia, hematoma, pseudoaneurysm, thromboembolism, tamponade, and red blood cell transfusion) and in-hospital death. For patients who experienced stroke after LB ablation, we investigated their discharge locations to estimate stroke severity.

### Statistical analyses

Categorical data are presented as frequencies (percentages). Continuous data are presented as medians (interquartile ranges [IQRs]). The Wilcoxon rank sum or Kruskal-Wallis test was used to compare continuous data. The χ^2^ or Fisher exact test was used to compare categorical data.

We performed propensity score (PS)–matching analysis separately to compare the in-hospital outcomes between LB, cryoballoon, and radiofrequency ablation procedures. Multivariable logistic regression models were used to calculate the PS, which represents the probability of undergoing LB ablation. Fifteen baseline characteristics were used as independent variables: age, sex, BMI, emergency hospitalization, paroxysmal AF, heart failure, ischemic heart disease, hypertension, diabetes mellitus, dyslipidemia, chronic kidney disease, dialysis, chronic obstructive pulmonary disease, previous stroke or TIA, and thromboembolism. Matching was performed using a nearest-neighbor matching algorithm (ratio 1:1 without replacement), with a caliper of width of 0.2 standard deviation of the logit of the estimated PS. The balance of each covariate between the 2 groups before and after matching was evaluated using standardized differences. The absolute value of the standardized difference of 10% was considered a relatively small imbalance. To estimate the odds ratios (ORs) and 95% confidence intervals (CIs) of LB ablation compared with radiofrequency or cryoballoon ablation, mixed-effects logistic regression analysis using the institute as a random intercept was performed for combined complications, in-hospital mortality, and complications. All statistical comparisons were 2-sided, and *P* < .05 was considered significant. All analyses were performed using STATA version 18.0 (StataCorp LLC).

## Results

### Baseline characteristics

In total, 179,207 patients were included in this study: those who underwent LB (n = 3193; median age 68 years; IQR 60–74 years), cryoballoon (n = 40,355; median age, 69 [60–75] years), or radiofrequency (n = 135,659; median age 69 years; IQR 61–75 years) ablation procedures (Table 1). During the study period, the number of LB ablation procedures was low, accounting for only 1.8% of all AF ablation procedures included in this study. LB ablation was performed at 98 facilities, with 3 of these accounting for 25% of all LB procedures. The proportion of male patients was higher in the LB ablation group than in the cryoballoon ablation group (70.7% vs 67.3%; *P* < .001) and comparable to the radiofrequency ablation group (70.7% vs 69.6%; *P* = .22). The proportion of patients with paroxysmal AF was higher in the LB ablation group than in the cryoballoon (86.5% vs 81.1%; *P* < .001) and radiofrequency ablation (86.5% vs 49.6%; *P* < .001) groups. The proportion of patients undergoing dialysis was lower in the LB ablation group than in the radiofrequency ablation group (0.9 % vs 1.4%; *P* = .01). The proportion of patients with a history of cerebral infarction or TIA was similar across the ablation groups. In Japan, most CA procedures for AF are performed in inpatient settings, and the median length of stay was 3 days (IQR 2–4 days) in the LB ablation group, which was similar to that in the cryoballoon or radiofrequency ablation groups.

**Table 1:** Baseline characteristics of the original cohorts

Characteristic	Laser balloon	Radiofrequency	Cryoballoon	*P*
	(n = 3193)	(n = 135,659)	(n = 40,355)	
Age (y)	68 (60–74)	69 (61–75)	69 (60–75)	<.001
Men	2256 (70.7)	94,457 (69.6)	27,157 (67.3)	<.001
BMI (kg/m^2^)	24.1 (21.9–26.3)	24.1 (21.9–26.6)	23.8 (21.6–26.2)	<.001
Emergency	30 (0.9)	2,575 (1.9)	650 (1.6)	<.001
Paroxysmal atrial fibrillation	2762 (86.5)	67,249 (49.6)	32,716 (81.1)	<.001
Heart failure	846 (26.5)	34,567 (25.5)	9,838 (24.4)	<.001
Ischemic heart disease	609 (19.1)	21,240 (15.7)	6,865 (17.0)	<.001
Hypertension	2499 (78.3)	110,929 (81.8)	30,626 (75.9)	<.001
Diabetes mellitus	575 (18.0)	27,409 (20.2)	8,011 (19.9)	.004
Dyslipidemia	1368 (42.8)	56,044 (41.3)	16,224 (40.2)	<.001
Chronic kidney disease (nondialysis)	115 (3.6)	3,767 (2.8)	657 (1.6)	<.001
Dialysis	28 (0.9)	1,928 (1.4)	692 (1.7)	<.001
Chronic obstructive pulmonary disease	85 (2.7)	3,405 (2.5)	803 (2.0)	<.001
Previous stroke or TIA	37 (1.2)	1,503 (1.1)	396 (1.0)	.09
Previous stroke	33 (1.0)	1,439 (1.1)	381 (0.9)	.13
Previous TIA	4 (0.1)	65 (0.05)	17 (0.04)	.12
Thromboembolism	0 (0.0)	51 (0.1)	168 (0.1)	0.14
CHA_2_DS_2_-VASc score	3 (2–4)	3 (2–4)	3 (2–4)	<.001
Length of stay (d)	3 (3–4)	3 (3–4)	3 (3–4)	<.001

Values are presented as median (interquartile range) or n (%).

BMI = body mass index; TIA = transient ischemic attack.

Among those who underwent LB ablation, 101 patients (3.2%) experienced periprocedural complications (Table 2). The incidence rate of cerebral infarction or TIA was 1.6%, relatively higher than the rates of other complications. The in-hospital mortality and readmission within 30 days of discharge were low (0.03% and 0.8%, respectively) in the LB ablation group.

**Table 2:** In-hospital outcomes after catheter ablation for atrial fibrillation

Outcome	Original cohort			PS-matched cohorts			*P*	
	Laser balloon	Radiofrequency	Cryoballoon	Laser balloon	Radiofrequency	Cryoballoon	Laser balloon vs radiofrequency	Laser balloon vs cryoballoon
	(n = 3193)	(n = 135,659)	(n = 40,355)	(n = 3193)	(n = 3193)	(n = 3193)		
Composite complication	101 (3.2)	3232 (2.4)	861 (2.1)	101 (3.2)	76 (2.4)	57 (1.8)	.06	<.001
In-hospital death	1 (0.03)	41 (0.03)	12 (0.03)	1 (0.03)	3 (0.1)	1 (0.03)	.32	>.99
Spasm of the coronary artery	0 (0)	60 (0.04)	27 (0.1)	0 (0.0)	2 (0.06)	1 (0.03)	.16	.32
Myocardial infarction	4 (0.1)	89 (0.1)	27 (0.1)	4 (0.1)	3 (0.1)	2 (0.1)	.71	.41
Sick sinus syndrome	7 (0.2)	542 (0.4)	96 (0.2)	7 (0.2)	10 (0.3)	7 (0.2)	.47	>.99
Complete atrioventricular block	2 (0.1)	50 (0.04)	20 (0.05)	2 (0.1)	2 (0.1)	2 (0.1)	>.99	>.99
Cardiac tamponade	17 (0.5)	852 (0.6)	188 (0.5)	17 (0.5)	27 (0.8)	21 (0.7)	.13	.52
Pneumothorax	8 (0.3)	374 (0.3)	109 (0.3)	8 (0.3)	7 (0.2)	4 (0.1)	.80	.25
Pneumonia	8 (0.3)	326 (0.2)	96 (0.2)	8 (0.3)	7 (0.2)	3 (0.1)	.80	.13
Thromboembolism	0 (0)	168 (0.1)	51 (0.1)	0 (0)	2 (0.1)	5 (0.2)	.16	.025
Stroke or TIA	50 (1.6)	514 (0.4)	141 (0.3)	50 (1.6)	14 (0.4)	9 (0.3)	<.001	<.001
Stroke	50 (1.6)	510 (0.4)	137 (0.3)	50 (1.6)	14 (0.4)	8 (0.3)	<.001	<.001
TIA	0 (0)	6 (0.004)	5 (0.01)	0 (0)	0 (0)	1 (0.03)	–	.32
Phrenic nerve palsy	3 (0.1)	29 (0.02)	28 (0.1)	3 (0.1)	0 (0)	1 (0.03)	.08	.32
Hematoma	3 (0.1)	196 (0.1)	62 (0.2)	3 (0.1)	5 (0.2)	2 (0.1)	.48	.65
Pseudoaneurysm	1 (0.03)	80 (0.1)	41 (0.1)	1 (0.03)	1 (0.03)	1 (0.03)	>.99	>.99
Red blood cell transfusion	15 (0.5)	685 (0.5)	175 (0.4)	15 (0.5)	13 (0.4)	9 (0.3)	.70	.22
Emergency readmission within 30 d of discharge	27 (0.8)	1144 (0.8)	300 (0.7)	27 (0.8)	22 (0.7)	19 (0.6)	.47	.24

Values are presented as n (%).

The laser balloon group originally comprised 3193 patients, all of whom were included in the 1:1 PS matching without replacement for both laser balloon vs radiofrequency and laser balloon vs cryoballoon comparisons. Consequently, each comparison included 3193 patients per group.

PS = propensity score; TIA = transient ischemic attack.

### The results of logistic analysis in PS-matched cohorts

**Figure 2:**
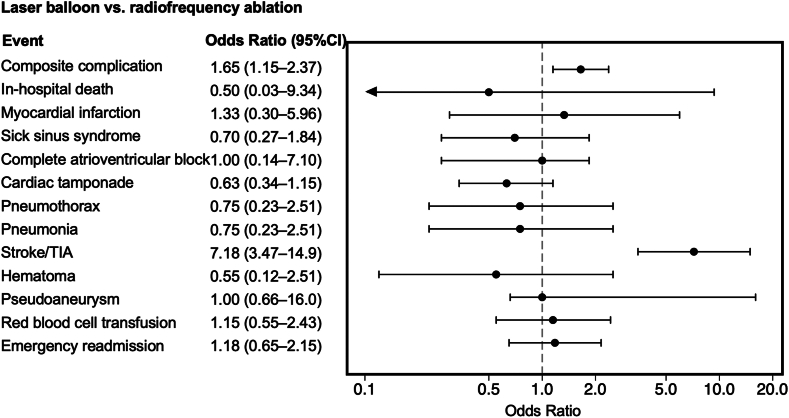
Results of logistic analysis in the propensity score–matched cohort comparing laser balloon and radiofrequency ablation procedures. CI = confidence interval; TIA = transient ischemic attack.

**Figure 3:**
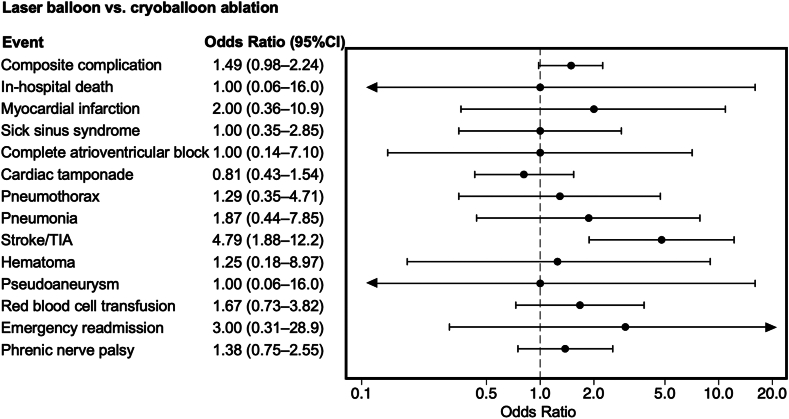
Results of logistic analysis in the propensity score–matched cohort comparing laser balloon and cryoballoon ablation procedures. CI = confidence interval; TIA = transient ischemic attack.

The LB group originally comprised 3193 patients, all of whom were included in 1:1 PS matching without replacement in both comparisons. Consequently, both LB ablation vs radiofrequency ablation and LB ablation vs cryoballoon ablation comparisons had 3193 patients per group (Table 3). The standardized differences in covariates in both PS-matched cohorts were <0.1, indicating a well-balanced comparison. As a result of mixed-effects logistic regression analysis in the PS-matched cohort, LB ablation was associated with a higher incidence of combined complications compared with radiofrequency ablation (3.2% vs 2.4%; OR 1.64; 95% CI 1.15–2.37) whereas no significant differences were observed between LB and cryoballoon ablation groups (3.2% vs 1.8%; OR 1.49; 95% CI 0.98–2.24) (Figures 2 and 3). In the comparison of individual complications, LB ablation was associated with a higher incidence of cerebral infarction compared with radiofrequency (1.6% vs 0.4%; OR 7.18; 95% CI 3.47–14.9; *P* < .001) or cryoballoon (1.6% vs 0.3%; OR 4.79; 95% CI 1.88–12.2; *P* = .01) ablation. In-hospital mortality, emergency readmission, and other complication rates did not differ according to the ablation system.

**Table 3:** Baseline characteristics of the PS-matched cohorts

Characteristic	Laser balloon ablation	Radiofrequency ablation	Cryoballoon ablation	Laser balloon ablation vs radiofrequency ablation		Laser balloon ablation vs cryoballoon ablation	
	(n = 3193)	(n = 3193)	(n = 3193)	|Std diff|	*P*	|Std diff|	*P*
Age (y)	68 (60–74)	69 (60–74)	68 (60–74)	0.005	.75	0.005	.80
Men	2256 (70.7)	2263 (70.9)	2341 (70.2)	0.005	.85	0.01	.68
BMI (kg/m^2^)	24.1 (21.9–26.3)	23.8 (21.7–26.4)	23.8 (21.8–26.2)	0.026	.09	0.023	.06
Emergency hospitalization	30 (0.9)	25 (0.8)	37 (1.2)	0.017	.50	0.022	.39
Paroxysmal atrial fibrillation	2762 (86.5)	2760 (86.4)	2773 (86.8)	0.002	.94	0.010	.69
Heart failure	846 (26.5)	847 (26.5)	876 (27.4)	0.001	.98	0.021	.40
Ischemic heart disease	609 (19.1)	588 (18.4)	630 (19.7)	0.017	.50	0.017	.51
Hypertension	2499 (78.3)	2462 (77.1)	2489 (78.0)	0.028	.27	0.008	.76
Diabetes mellitus	575 (18.0)	565 (17.7)	555 (17.4)	0.008	.74	0.016	.51
Dyslipidemia	1368 (42.8)	1359 (42.6)	1347 (42.2)	0.006	.82	0.013	.60
Chronic kidney disease (nondialysis)	115 (3.6)	117 (3.7)	115 (3.6)	0.003	.89	<0.001	>.99
Dialysis	28 (0.9)	26 (0.8)	39 (1.2)	0.007	.78	0.034	.18
Chronic obstructive pulmonary disease	85 (2.7)	78 (2.4)	95 (3.0)	0.014	.58	0.019	.45
Previous stroke or TIA	37 (1.2)	37 (1.2)	38 (1.2)	<0.001	>.99	0.003	.91
Previous stroke	33 (1.0)	36 (1.1)	37 (1.2)	0.009	.72	0.012	.63
Previous TIA	4 (0.1)	1 (0.03)	2 (0.1)	0.034	.18	0.020	.41
Thromboembolism	0 (0.0)	2 (0.1)	5 (0.2)	0.027	.16	0.017	.025

Values are presented as median (interquartile range) or n (%).

The laser balloon group originally comprised 3193 patients, all of whom were included in the 1:1 PS matching without replacement for both laser balloon vs radiofrequency and laser balloon vs cryoballoon comparisons. Consequently, each comparison included 3193 patients per group.

BMI = body mass index; PS = propensity score; Std diff = standardized difference; TIA = transient ischemic attack.

### Discharge location of patients who experienced stroke after LB ablation

Of the 50 patients in the original cohort who experienced stroke after LB ablation, 43 (86%) were discharged whereas 7 (14%) were transferred to other hospitals. The median length of stay in hospital among patients who experienced stroke was 3 days (interquartile range 3–11 days).

## Discussion

In this study, we used a large-scale nationwide claims database to analyze perioperative complications, including the largest number of LB ablation cases. As most AF ablation procedures in Japan are performed during hospitalization, this allowed a comprehensive analysis of immediate postoperative complications.

The major findings of the present study are as follows: (1) the number of LB ablation procedures was limited, accounting for only 1.8% of CA for AF; (2) in-hospital complications occurred in 3.2% of the LB ablation group, and stroke was the most common complication, occurring in 1.6% of patients; (3) LB ablation was associated with a higher risk of complications compared with radiofrequency ablation; (4) LB ablation was associated with a higher risk of stroke compared with cryoballoon or radiofrequency ablation; (5) no significant differences in in-hospital mortality, other complications, or emergency readmission within 30 days of discharge were observed between LB and cryoballoon ablation procedures or between LB and radiofrequency ablation procedures.

Stroke was the most common periprocedural complication in the LB group, occurring in 1.6% of patients; this incidence was comparable to that reported in previous reports, which ranged from 0% to 4.4%.^3^, ^4^, ^5^^,^^7^, ^8^, ^9^^,^^22^ A prospective, multicenter, observational study involving 304 LB ablation cases in Japan reported a stroke rate of 1.0%, which is consistent with our findings.^23^

Furthermore, the incidence rate of procedure-related stroke or TIA after radiofrequency or cryoballoon ablation in the present study was consistent with earlier reports.^18^^,^^21^^,^^24^^,^^25^ Since postprocedural brain magnetic resonance imaging is not the standard of care, almost all the events reported here are likely to have been symptomatic.

In Japan, it is common for patients to be transferred to a rehabilitation hospital after a stroke if active rehabilitation is expected to improve their physical function. However, in this study, among the 50 patients who developed stroke after LB ablation, only 14% were transferred to a hospital whereas the remaining 86% were discharged home. This suggests that the symptoms associated with stroke are relatively mild or, even if some symptoms persist, home-based rehabilitation is sufficiently feasible.

Asymptomatic cerebral lesions after LB ablation occur in 11%–24% of cases,^7^^,^^26^ a rate comparable to that observed after radiofrequency ablation (2.7%–24%) or cryoballoon ablation (5.2%–18%).^7^^,^^27^^,^^28^ The mechanism underlying stroke occurrence after LB ablation is not fully understood. However, laser energy deployment adjacent to blood can theoretically increase the risk of thermocoagulation with subsequent thromboembolism.^8^ In addition, microthrombus formation resulting from endothelial injury caused by laser energy may play a role. Furthermore, the LB, similar to the cryoballoon, is a device with a deflectable balloon structure, which may predispose to air embolism due to air trapped around the balloon. Further studies are required to elucidate these underlying mechanisms.

Cardiac tamponade and phrenic nerve palsy are other possible complications after LB ablation.^3^ In this study, the incidence rate of cardiac tamponade was 0.5%, which was comparable to that reported in previous studies (0%–2.0%),^3^, ^4^, ^5^^,^^8^, ^9^, ^10^, ^11^ and no significant association with LB use was observed. In contrast, the incidence rate of phrenic nerve palsy in the LB ablation group was 0.1%, which was relatively smaller than that reported in previous reports (1.3%–6.0%).^3^, ^4^, ^5^^,^^8^, ^9^, ^10^, ^11^, ^12^^,^^23^ The phrenic nerve runs anterior to the right atrium; thus, the use of an LB during right-sided PVI carries the risk of phrenic nerve palsy. However, previous reports have shown that in most cases, except for a few, phrenic nerve palsy is transient and resolves over time.^3^, ^4^, ^5^^,^^8^, ^9^, ^10^, ^11^, ^12^ In the present study, it is possible that only severe or persistent cases were captured through diagnostic coding.

This study has some limitations. First, the study design was observational and retrospective and the treatments were not randomized. Second, because DPC data were based on medical claims, data that were not directly related to costs were not completely validated. Claims-based data, such as DPC data, may not fully capture transient or clinically minor complications. However, previous studies have proven the validity of the diagnoses in the JROAD-DPC database in comparison with other nationwide databases or in-hospital registries.^15^^,^^29^ Characteristics of operators, appropriate or inappropriate therapy, and precise clinical information, such as laboratory or echocardiographic data, were not available. We were able to identify complications only during hospitalization; therefore, this study did not include long-term complications, such as pulmonary vein stenosis. However, the median length of stay was 3 days, which was sufficiently long to capture most periprocedural complications that occurred during the early postoperative period. In addition, although we used robust statistical methods to account for differences between the groups, we cannot rule out the possibility of residual confounding factors. Finally, pulsed-field ablation was not available in Japan during the study period; therefore, we were unable to conduct a comparative study.

## Conclusion

To date, this study includes the largest number of patients who underwent AF ablation with LB. Analysis of a nationwide database that includes >800 hospitals in Japan revealed that LB ablation accounted for only 1.8% of all AF ablation procedures, suggesting that it has not yet been widely adopted. Compared with radiofrequency ablation, LB ablation was associated with a higher risk of periprocedural complications. In particular, LB ablation was associated with a higher incidence of stroke than that with either radiofrequency or cryoballoon ablation, although no significant association was observed with in-hospital mortality or cardiac tamponade. Although this was observed using a large nationwide database, validation using other independent data sets is preferable.

## Appendix Supplementary Data

TableS1
