# Supplementary material for: Periprocedural complications after laser balloon ablation procedures for atrial fibrillation: An analysis using a nationwide claims database
Source: Heart Rhythm O2. 2025 Jul 29;6(10):1516–23. doi: 10.1016/j.hroo.2025.07.016 (PMC12570194; doi:10.1016/j.hroo.2025.07.016)
Supplement: TableS1 [file mmc1.docx]

**Supplementary Table 1. Code book**

^*1^ICD-10 codes unless otherwise specified. A code ending with “$” indicates inclusion of all values that begin with the preceding characters. The operation, reimbursement, and detailed diagnostic codes are the Japan-specific original codes.

| **Variable** | **Codes^*1^** |
| --- | --- |
| Catheter ablation | Operation codes: K595-00 and reimbursement code: 150346710 |
| Cryoballoon ablation | Reimbursement code: 710010840 |
| Laser balloon ablation | Reimbursement code: 710010950 excluding hot balloon identified by a device name |
| Atrial fibrillation | I480, I481, I482, I489 |
| Paroxysmal atrial fibrillation | Detailed diagnosis codes: 8846942, 98847818 |
| Other arrhythmias | I456, I47$, I483, I484, I4892, I490, I491, I492, I493, I4940, I4949, I498, I499 |
| Heart failure | I0981, I110, I130, I132, I5020, I5021, I5022, I5023, I5024, I5025, I5026, I5027, I5028, I5029, I503, I504, I505, I506, I507, I508, I509, I97131, |
| Ischemic heart disease | I20$, I21$, I22$, I23$. I24$, I25$ |
| Chronic kidney disease | K767, K289, R392, N18$, N19$ |
| Chronic obstructive pulmonary disease | J4$ |
| Stroke | I63$ |
| Transient ischemic attack | G45$ |
| Thromboembolism | H342, I24$, I269, I740, I741, I744, I748, I749, K550, K868, N280, T790 |
| Spasm of the coronary artery | I201 |
| Sick sinus syndrome | I495, I455 |
| Complete atrioventricular block | I442 |
| Myocardial infarction | I21$, I22$, I23$ |
| Phrenic nerve palsy | G588 |
| Pneumothorax | J159, J189, J690, J942 |
| Pneumonia | J159, J189, J690 |
| Hematoma | S701, S801, T140, T810 |
| Pseudoaneurysm | I724 |
| Cardiac tamponade | I319, I971, J985, procedure codes: J048-00, K539-00 |
| Blood transfusion | Operation codes: K920, K9201–K9205 |
